# Spectroscopic and Electrical Insights into Humidity-Induced Modifications of Graphene Structures

**DOI:** 10.1021/acsomega.5c10687

**Published:** 2026-06-02

**Authors:** Dinara Sobola, Pavel Kaspar, Nikola Papež, Rashid Dallaev, Vladimír Holcman, Zdenka Fohlerová, Petr Sedlák, Mohammed Ahmed Al-Anber, Robert Macků

**Affiliations:** †, Institute of Physics of Materials, Czech Academy of Sciences, South Moravian Region, Brno, CZ 616 00, Czech Republic; ‡ 48274 Brno University of Technology, Faculty of Electrical Engineering and Communication, Department of Physics, South Moravian Region, Brno, CZ 616 00, Czech Republic; § Brno University of Technology, Faculty of Electrical Engineering and Communication, Department of Microelectronics, South Moravian Region, Brno, CZ 616 00, Czech Republic; ∥ Applied Science Private University, Faculty of Sciences, Department of Chemistry, Amman 11931, JO, Jordan; □ Institute of Scientific Instruments, Czech Academy of Sciences, South Moravian Region, Brno, CZ 612 00, Czech Republic

## Abstract

This paper explores the environmental sensitivity of monolayer graphene-based sensor devices for use as sensors under controlled humidity conditions using a combination of scanning electron microscopy (SEM), Raman spectroscopy, Fourier transform infrared spectroscopy (FTIR), and Kelvin probe force microscopy (KPFM). The structural and electrical responses of the graphene devices were examined before and after exposure to water vapor. Raman spectroscopy confirmed the presence of minor defects localized near the contacts, while FTIR revealed distinct spectral changes associated with the adsorption of H_2_O and displacement of preadsorbed CO_2_. KPFM mapping demonstrated surface potential redistribution and a reduction of the work function, consistent with improved carrier mobility upon water adsorption. Electrical measurements showed resistance variations that diverge from theoretical predictions for pristine graphene, indicating that environmental contaminants strongly influence device behavior. These results highlight the dual role of water as both a contaminant stabilizer and a charge redistribution agent, emphasizing the importance of environmental control for graphene-based sensing applications, or the inclusion of the expectations of the shift of electric properties caused by the environmental contaminants.

## Graphical abstract

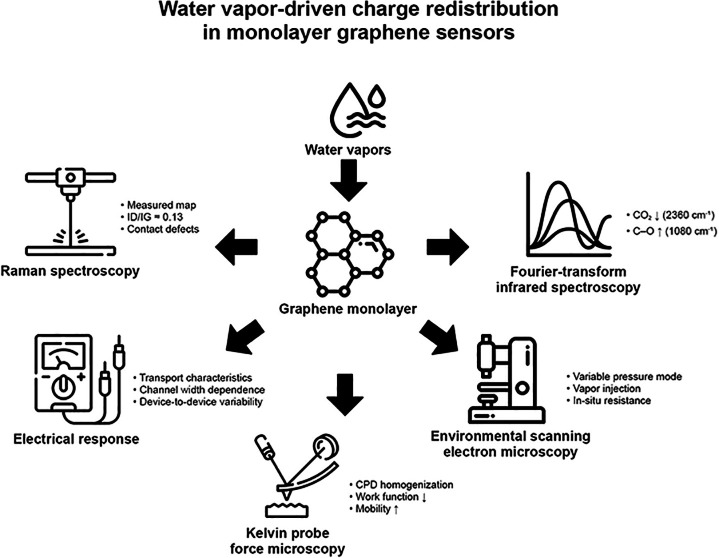

## 1 Introduction

Graphene has attracted sustained interest due to its exceptional electrical, mechanical and chemical properties, and, in particular, for its ability to transduce surface adsorption phenomena into changes in conductivity. Graphene is particularly attractive as a channel material, because its atomically thin structure and delocalized π-electron network confer an exceptionally high carrier mobility and sensitivity to surface charge perturbations.^1,2^ Its two-dimensional geometry means that the entire conduction channel lies at the surface, making conductivity extremely responsive to adsorption or chemical modification near the interface.^3^ These properties underpin the utility of graphene for sensors, where small change in surface chemistry, such as due to water or gas adsorption, can translate into a detectable change in electrical response. At the same time, graphene can be integrated into flexible, low-volume devices and supports a wide dynamic range of carrier density via field-effect gating, facilitating both fundamental studies and practical transistor and sensor applications.^4^ Graphene-based sensors have therefore been widely investigated as sensing platforms, since even trace amounts of adsorbed oxygen, carbon dioxide or water can noticeably alter charge transport, contact behavior and work function.^5–8^ Humidity plays a particularly significant role, because adsorbed water interacts with graphene through two pathways: physisorption that displaces preadsorbed gases such as CO_2_, and surface oxidation at defect or functional sites that produces OH and C–O groups. These mechanisms redistribute surface charge and modify the local electrostatic potential, resulting in measurable shifts in work function and mobility.^9^ Theoretical treatments for pristine graphene typically predict that molecular adsorption increases carrier scattering and resistance. However, practical devices contain fabrication-induced defects and environmental adsorbates that can be stabilized or passivated by water, leading instead to partial restoration of conductivity.^10^ This disagreement arises because idealized pristine-surface models neglect unavoidable contamination. To bridge this gap, we propose a contamination-aware model that considers the complex environment where water and other species are continuously exchanging with the surface. As a consequence, humidity-induced resistance changes are often nonmonotonic and may contradict expectations based on pristine-surface theory. Understanding this behavior requires experimental approaches that resolve both the chemical identity of the adsorbates and the electrostatic and electrical consequences of their interaction with graphene. In this work, we investigate the effects of water vapor adsorption on monolayer graphene sensor devices operated in a chemiresistive sensing configuration. SEM confirms structural integrity; Raman spectroscopy provides information on disorder; FTIR identifies chemical changes related to adsorbate displacement and oxidation; and KPFM maps shifts in surface potential and work function. Electrical measurements are performed under both vacuum-cleaned (pristine-like) and ambient conditions to compare idealized models with contaminated device behavior.

Recent advances in nanomaterial-enabled sensing platforms demonstrate that low-dimensional materials support diverse transduction mechanisms beyond conventional resistive and capacitive humidity detection. Nanostructured metal oxides, 2D materials, and hybrid composites have achieved high sensitivity by enhancing surface adsorption and proton conduction pathways,^11–14^ while self-powered and nanoengineered architectures enable low-power and flexible operation.^15,16^ In parallel, optoelectronic studies of nanostructured and 2D systems have demonstrated strong carrier modulation via interfacial charge transfer, defect engineering, and field-enhanced light–matter interactions.

While most humidity-sensing approaches rely on macroscopic conductance or capacitance changes governed by physisorbed water layers, proton hopping, or photocarrier modulation, these techniques primarily provide global electrical readouts without direct chemical identification of the interfacial processes involved. Surface-sensitive methods such as Kelvin probe force microscopy (KPFM) have enabled spatial mapping of humidity-induced work-function variations and dynamic potential propagation,^17–19^ yet they generally lack simultaneous spectroscopic identification of displaced or adsorbed species and do not directly correlate local charge redistribution with macroscopic transport under controlled versus ambient conditions.

In this work, we address this gap by combining *I*–*V* characteristics with Raman and FTIR-based chemical analysis and KPFM mapping to establish a multimodal chemical–electrical correlation framework. We demonstrate that water adsorption can displace specific airborne adsorbates from graphene, locally enhancing carrier mobility, while simultaneously generating oxidation-related trap states that introduce hysteresis and nonmonotonic resistance changes. By experimentally linking which molecules are exchanged at the surface (FTIR), where charge redistribution occurs (KPFM), and how device resistance evolves in pristine-like and environmentally exposed samples, we provide a chemically resolved interpretation of adsorption-mediated carrier fluctuations under dark conditions. We therefore address how water adsorption modifies graphene surface chemistry, including displacement of preadsorbed species, how these chemical changes alter local work function and charge distribution and how these effects translate into macroscopic transport behavior. We hypothesize that water simultaneously enhances local mobility by replacing strongly binding adsorbates while introducing oxidation-induced trap states, and that the interplay between these mechanisms accounts for the contrasting resistance behavior observed experimentally.

## 2 Materials and Methods

The experiments were conducted using monolayer graphene-based sensor chip, sourced from Sigma-Aldrich (product code GRFETS10). These sensors utilize chemical vapor deposition (CVD)-grown graphene as the active material. The sensors can be used as a field effect transistor (FET) with a 90 nm thick SiO_2_ layer serving as the gate oxide, and the substrate has a resistivity ranging from 1 to 10 Ω·cm. The metallization consists of a 140 nm thick layer of nickel and aluminum.

The graphene used in these devices exhibits a high field-effect mobility of over 1000 cm^2^/V·s and a low residual charge carrier density of less than 2 × 10^12^ cm^–2^. The Dirac point is typically within the range of 10 to 40 V. These devices are robust, with a yield exceeding 75%, a maximum gate-source voltage rating of ± 50 V, and a thermal stability limit of up to 150 °C. The maximum allowable current density through the drain-source channel is 10^7^ A/cm^2^, making these structures suitable for a wide range of sensing applications.

A FEI Quattro SEM microscope was used to measure the response of the graphene channel to the gaseous environment. The vacuum system of this device is based on shared pumping of the column and chamber by a turbomolecular pump (TMP). Once a sufficient level of vacuum is achieved, the chamber is isolated by a proportional valve (CIV). At the same time, this instrument can operate in a low-vacuum mode (CIV is used for regulation) and with external gas saturation. In practical terms, this means that constant pumping controlled by CIV is maintained while water vapors are admitted from an external container into the chamber. The pressure in the chamber is regulated by a combination of a vacuum gauge (HVG) and a needle valve (NVC). A schematic of the environmental SEM-ESEM experimental configuration, including the pumping system, proportional isolation valve (CIV), external water vapor inlet and pressure control, is shown in Figure 1.

**Figure 1:**
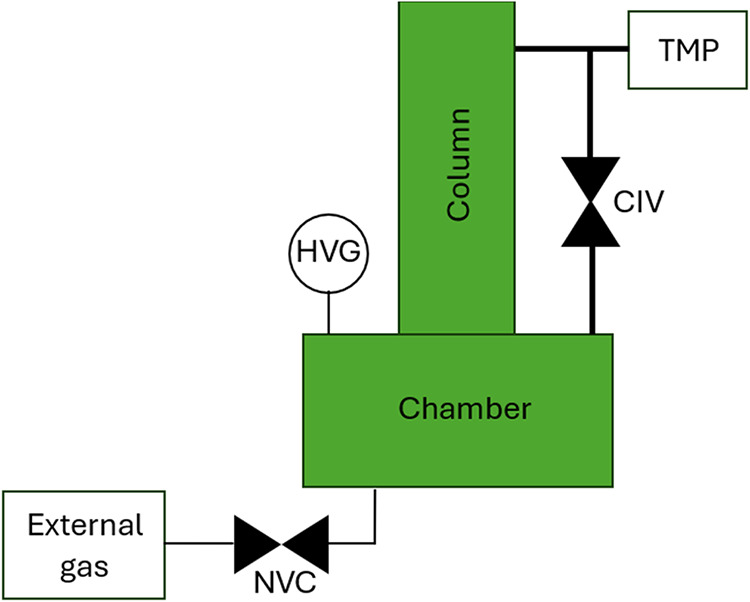
Schematic of the SEM–ESEM experimental setup used for humidity-controlled electrical measurements. The configuration includes a turbomolecular pumping system, proportional isolation valve (CIV), controlled water vapor inlet regulated by a needle valve controller (NVC), chamber pressure monitoring, and the positioning of the graphene device inside the SEM chamber for in situ resistance measurements under low-vacuum ESEM conditions.

Graphene chip structures were placed inside the SEM chamber for resistance measurements under controlled environments. During this measurement, RH was gradually increased from high-vacuum conditions (0.16% RH) to 18% RH, while both the step increments and exposure times were adjusted in accordance with the pressure. Testing was conducted in a water vapor atmosphere using the ESEM mode of SEM. In this low-pressure environment, the exposure conditions are defined by chamber pressure and vapor presence rather than by relative humidity, which is not directly applicable under ESEM operating conditions. The chamber environment was stabilized prior to data acquisition and stayed constant during each measurement. The electron beam operated at 5 kV and 14 pA for surface cleaning and imaging. Variations in the graphene resistance were monitored and recorded as a function of the experimental conditions to assess the material’s sensitivity to environmental changes.

Raman spectroscopy was conducted to investigate the structural characteristics of the samples using a WITec alpha300 R system (WITec, Ulm, Germany). The system operated with a 532 nm excitation wavelength and a laser power of 20 mW. The Raman signal was measured with an integration time of 25 s without accumulations. A Zeiss EC Epiplan-Neofluar Dic 50× objective was employed, and the grating used was G1 with 600 grooves per millimeter.

Fourier-transform infrared spectroscopy was performed using a Fourier infrared spectrometer coupled with an infrared microscope (Hyperion 3000 KIT, Bruker, Billerica, MA, USA). Measurements were carried out in reflection mode by 15x objective with a spectral resolution of 4 cm^–1^. Each spectrum was acquired by averaging 512 scans to improve the signal-to-noise ratio.

Kelvin Probe Force Microscopy (KPFM) measurements were performed using the NTEGRA Prima scanning probe microscope (NT-MDT Spectrum Instruments, Sutton 11A, 7327 AB Apeldoorn, The Netherlands). The measurements were carried out in semicontact SPM mode with the Kelvin probe technique, using the Nova software (version 1.1.1.16703). The lift height was set to 10 nm, generator amplitude of 0.7 V and an AC bias of 0 mV was applied at the resonance frequency of 227.075 kHz. The DC bias was automatically adjusted by the feedback loop to nullify the electrostatic force between the tip and the sample and to obtain the contact potential difference (CPD). The environmental conditions during the experiments were maintained at a relative humidity of 59.52% and a temperature of 21.83 °C. A HA-NC/Au probe (length 94 μm, width 34 μm, force constant 12 N/m) with a 20–30 nm gold coating on the tip side was used.

The contact potential difference is related to the work function difference between the tip and the sample. Calculation procedure is described in the section of Results and Discussion section dealing with KPFM. The KPFM analysis was focused on relative CPD variations and spatial trends induced by humidity exposure rather than on absolute work function values. Therefore, no external absolute tip calibration was performed. All comparative measurements were carried out using the same probe and identical acquisition parameters to ensure internal consistency of the CPD contrast.

Electrical characterization of the graphene-based sensor was performed using a parameter analyzer (4200A-SCS, Keithley, Solon, United States) coupled with a probe station (MPS150, Cascade Microtech, Beaverton, United States). The back gate was not actively biased during humidity measurements, and the device was operated in a two-terminal chemiresistive mode. The applied bias voltage was swept from −1 V to +1 V at a constant sweep rate. To ensure repeatability and stability, identical measurements were conducted on multiple sensors with the same structure and fabrication parameters, and a double sweep (forward and backward) was performed during each measurement to verify sweep reproducibility and to evaluate possible hysteresis effects. All electrical measurements were carried out under ambient laboratory conditions (air, 25 °C).

This regeneration procedure was applied after each measurement cycle and consistently restored the baseline resistance, confirming the reversible nature of the humidity-induced response. This procedure is referred to as the “clean” condition in the following text.

For vapor exposure experiments, the device was positioned 2 cm above the surface of demineralized water maintained at 80 °C, and exposed to the resulting water vapor for 90 s. This approach provided a high-humidity exposure (RH > 95%) but did not allow precise control or calibration of relative humidity. After exposure, the sensor was allowed to stabilize for 30 min (average cooling rate 2 °C/min) under ambient conditions (RH ≈ 50%) before the electrical response was recorded.

## 3 Results and Discussion

Several characterization methods have been employed to provide an overall picture on the properties of the graphene sensor, like SEM, Raman spectroscopy and FTIR before and throughout the development by interaction with atmospheric and water, starting with structural measurements to establish the baseline form and dynamic methods, such as KPFM and conductivity measurements.

Images were taken by scanning electron microscopy (SEM) to provide a clear idea about the setup of the sample active location (Figure 2). The black area shows the graphene layer, whereas the white areas display the Ni/Al contacts around them, with the gray area representing the nonparticipating areas of the substrate without electric activity. The measurement is done between the two larger, slanted contacts in the top left and bottom right corner. The thinner side contacts allow for different forms of interconnection, but in this care are not used at all.

**Figure 2:**
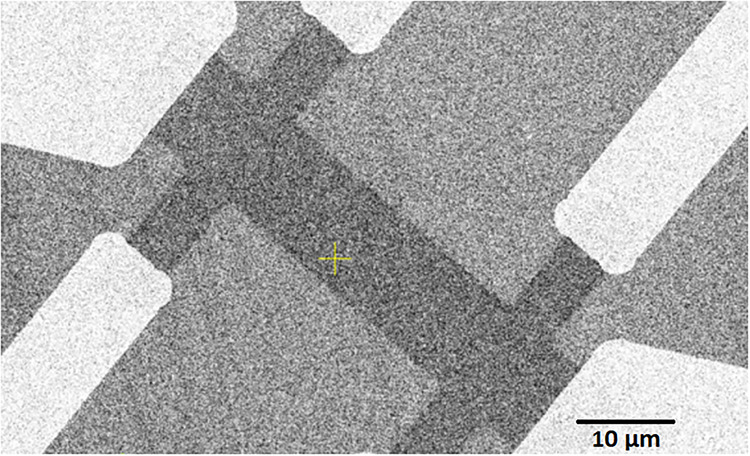
SEM image of the tested sensor.

The SEM images also confirm the structural integrity of the sensor, showing no significant damage or disruption to its framework. Even though some disorder has been observed near the contacts and is discussed in the Raman spectroscopy section of this paper (Figure 3b), the overall sensor remains intact and fully functional.

**Figure 3:**
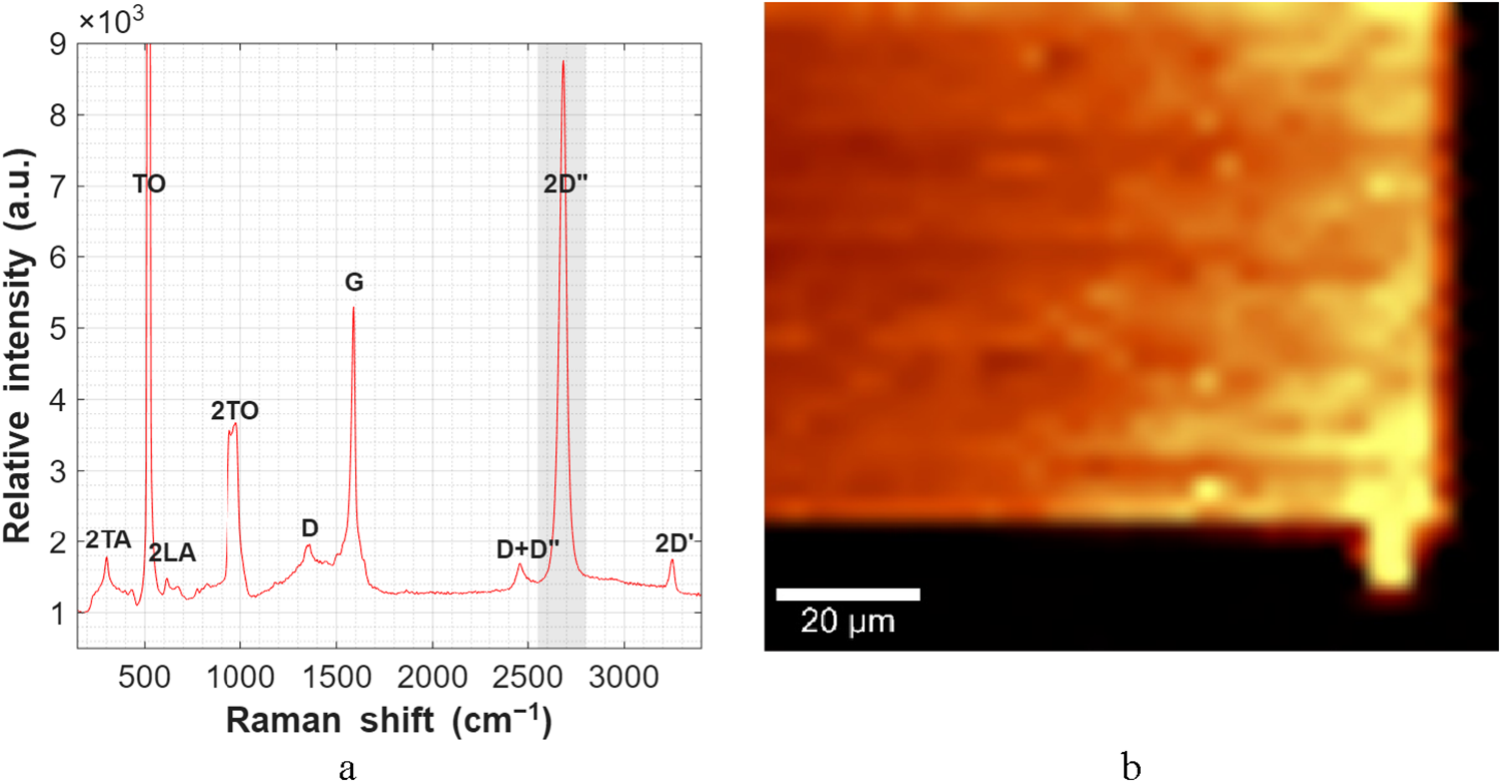
(a) Raman spectra and (b) map of the 2680 cm^–1^ peak distribution.

Raman spectroscopy was performed to establish information about the composition and quality of the graphene sensor. The results obtained show the typical spectrum of a single layer graphene^20^ with signal from silicon dioxide substrate. Figure 3a. shows that the general presence of the spectrum peaks is consistent with those reported in previous publications,^21,22^ whereas Figure 3b shows the spatial distribution of the 2D-band intensity. The relatively low contrast of the map is consistent with the monolayer thickness of graphene and the influence of the SiO_2_ substrate, which limits absolute Raman intensity variations. The mapping therefore primarily serves to confirm spatial uniformity of the graphene layer and the absence of large-scale structural degradation.

To provide a quantitative assessment of graphene quality and defect density, numerical peak analysis was performed on the measured Raman spectra after baseline correction. The extracted intensity ratio *I*_D_/*I*_G_ was approximately 0.13, indicating a low density of structural defects in the graphene channel. The *I*_2D_/*I*_G_ ratio was approximately 2.0, which is characteristic of monolayer graphene. The G and 2D peak positions were centered at 1586 cm^–1^ and 2682 cm^–1^, respectively. The corresponding full width at half-maximum (FWHM) values were approximately 32 cm^–1^ for the G peak and 43 cm^–1^ for the 2D peak, consistent with high-quality CVD-grown monolayer graphene. A localized increase of the D-band intensity was observed near the metal contact regions, indicating fabrication-induced disorder confined mainly to the contact vicinity.

Fourier transform infrared spectroscopy is more capable of detecting the presence of atmospheric gases,^23^ so it has been employed to better display the changes in adsorbed gaseous molecules. The most dominant feature of the FTIR (Figure 4) spectrum is the double peak at around 2360 m^–1^. This peak is most commonly attributed to atmospheric CO_2_, which makes it an artifact or otherwise undesirable peak in the displayed spectrum.^24^ In this case, however, a difference in the magnitude of this peak is visible between the sensor with and without H_2_O. The CO_2_ peak here represents the adsorbed gases onto the surface of the sensor. It shows an approximate 76% reduction in magnitude in the version with bound H_2_O vapor, in comparison to the version without it, which suggests that the water vapor adsorbs onto the same positions of sample, replacing the adsorbed CO_2_. This also means that in order for the sensor to reset itself and be capable of a new detection cycle, the CO_2_ would need to be properly removed, otherwise the sensor would not be able to perform in its full capacity. The second most prominent peak at around 1080 m^–1^ is attributed to a stretching vibration of the C–O (alkoxy)^25^ bond of the graphene layer, which is usually expected to diminish after reduction reaction.^26^ From an electrochemical standpoint, in humid air the adsorbed water can participate in a redox cycling at graphene oxide defects, which results in −OH groups bonding onto the surface in a surface oxidation process, resulting in slightly higher conductivity afterward.^27,28^ Therefore, the C–O peak growing by about 52% on this water-induced oxidation is to be expected. This phenomenon is *reversible*, as annealing under vacuum or electron-beam irradiation restores the sensor to its initial state without any observable degradation of its electrical characteristics.

**Figure 4:**
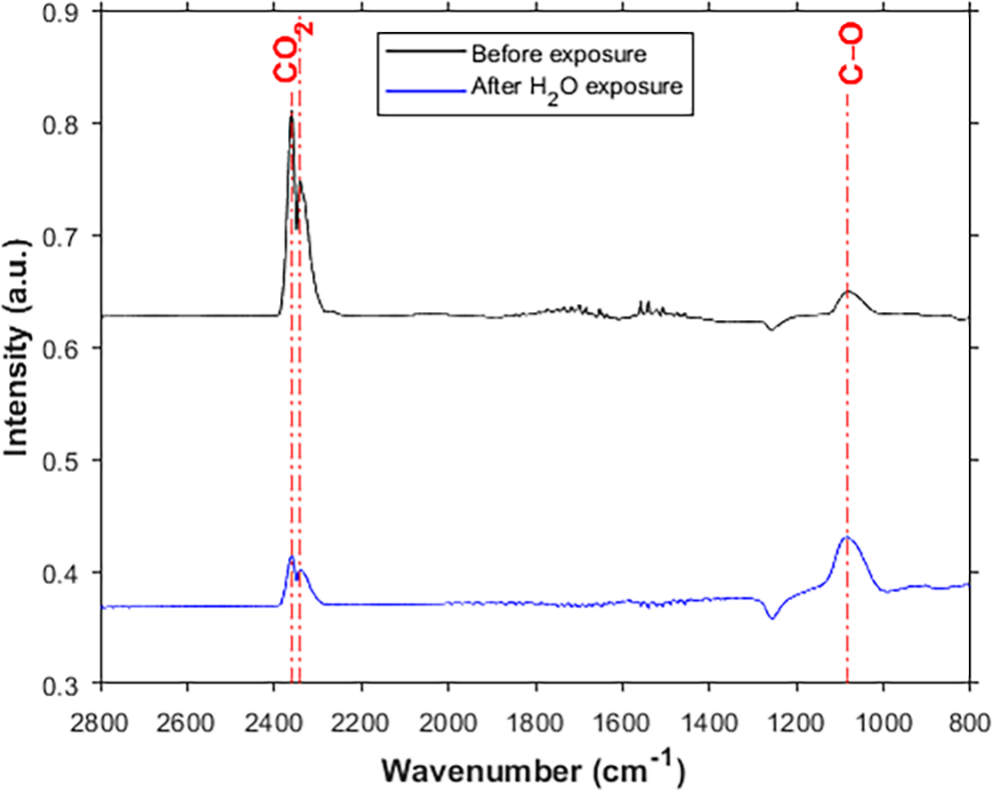
FTIR spectrum of the sample before and after H_2_O exposure.

Kelvin Probe Force Microscopy (KPFM) has been employed to display the distribution of surface potential, work function maps and carrier density before and after exposure to water. Figure 5 shows the changes in distribution of surface potential, where after water exposure, contact potential difference (CPD) increases over the surface of the sample, which shows the charge redistribution and increased conductivity^29,30^ as was inferred from FTIR.

**Figure 5:**
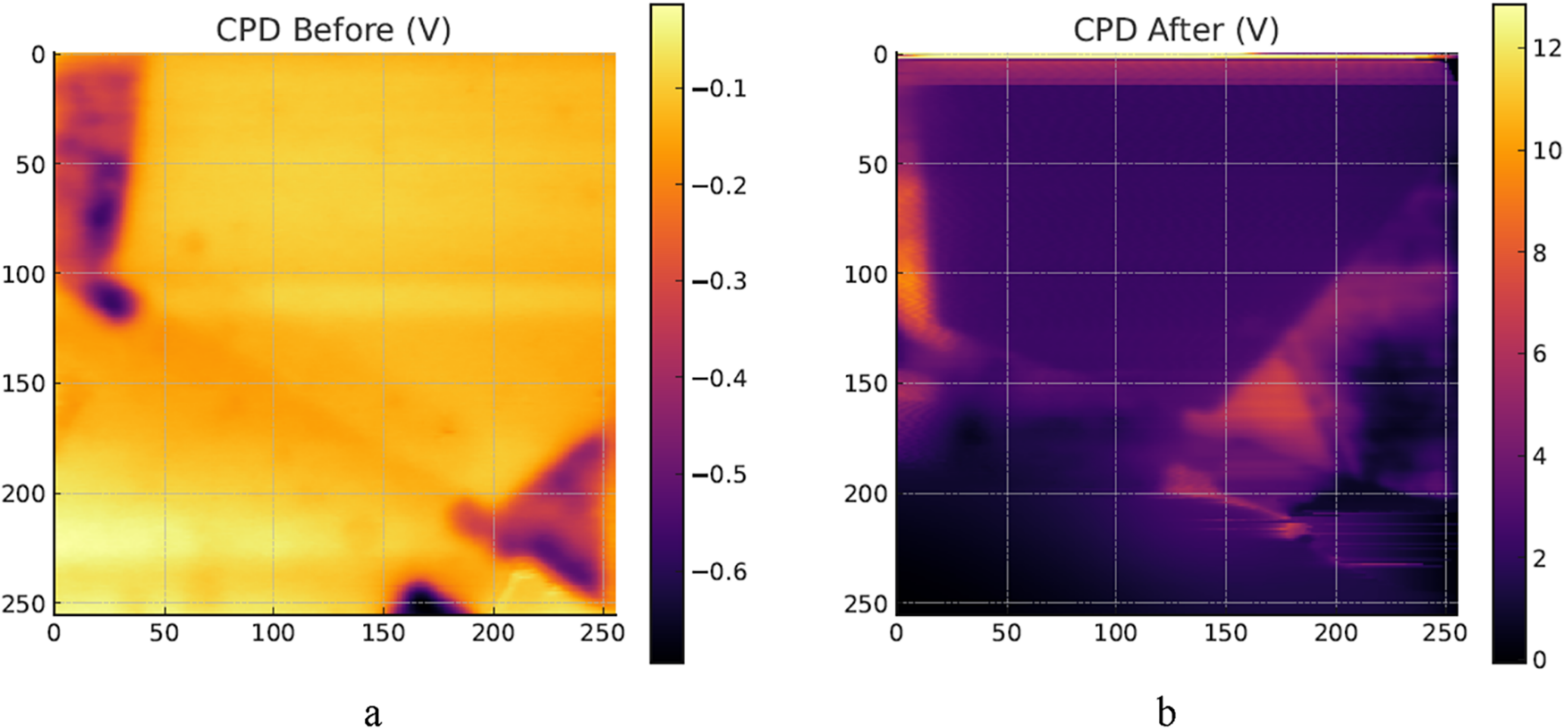
KPFM displaying surface potential (CPD) maps (a) before and (b) after water exposure.

It is possible to gain information about the work function by calculating

1 $$\phi_{\mathrm{sample}}=\phi_{\mathrm{tip}}-e\times V_{\mathrm{CPD}}$$

where ϕ_tip_ is the work function of the probe tip used for the measurement, *e* is the elementary charge of an electron and *V*_CPD_ is the measured contact potential difference.^31^ The shift shows how water adsorption changes electron energy levels (Figure 6). As the surface is crowded with other adsorbates, aside from water, the decrease of work function after water exposure helps the carrier mobility, and points toward the possibility of water adsorption replacing some of the other contaminants,^32^ which pushes the electric properties more toward those of pure graphene, even if not by huge amounts.

**Figure 6:**
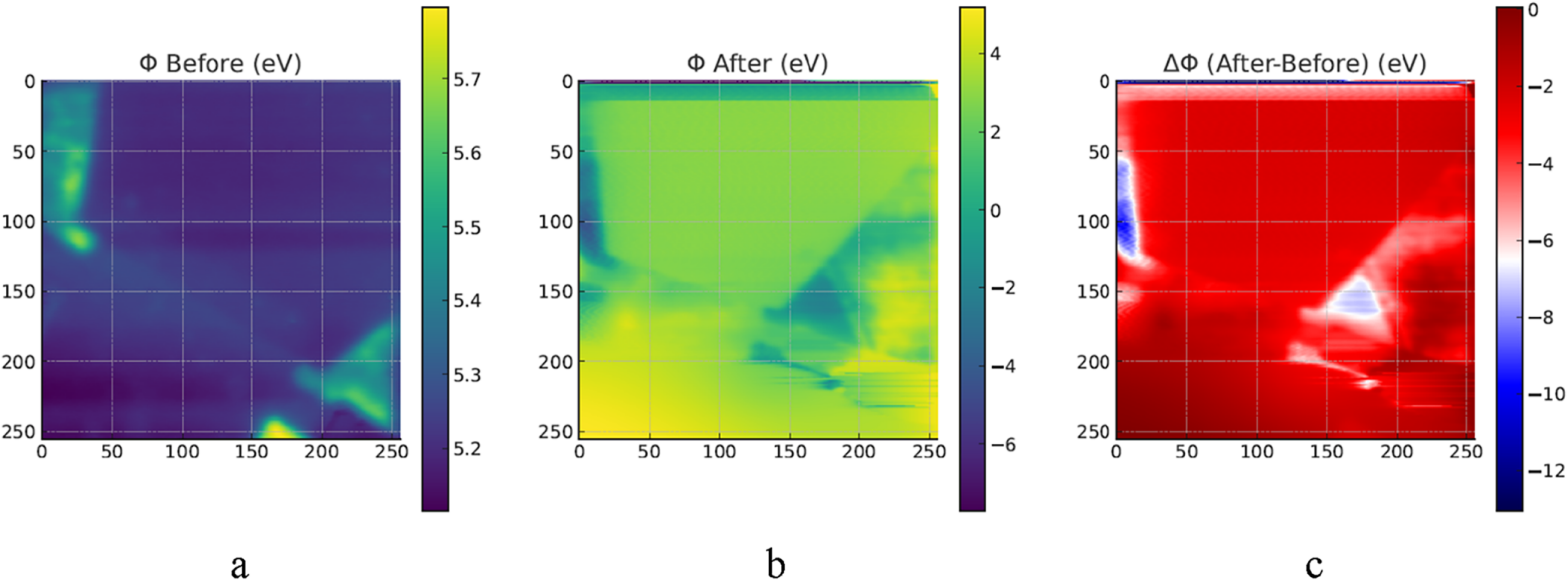
KPFM displaying work function maps (a) before and (b) after water exposure, and (c) their change.

By utilizing KPFM it is also possible to calculate (eq 2) and map out the spatial gradient ∇*V* of an electric field (Figure 7), where the bright regions indicate abrupt changes in local potential^33^

2 $$\begin{array}{l} \nabla V\left(x,y\right)=\left(\frac{\partial V}{\partial x},\frac{\partial V}{\partial y}\right)\\ |\nabla V|=\sqrt{{\left(\frac{V}{x}\right)}^{2}+{\left(\frac{V}{y}\right)}^{2}} \end{array}$$

**Figure 7:**
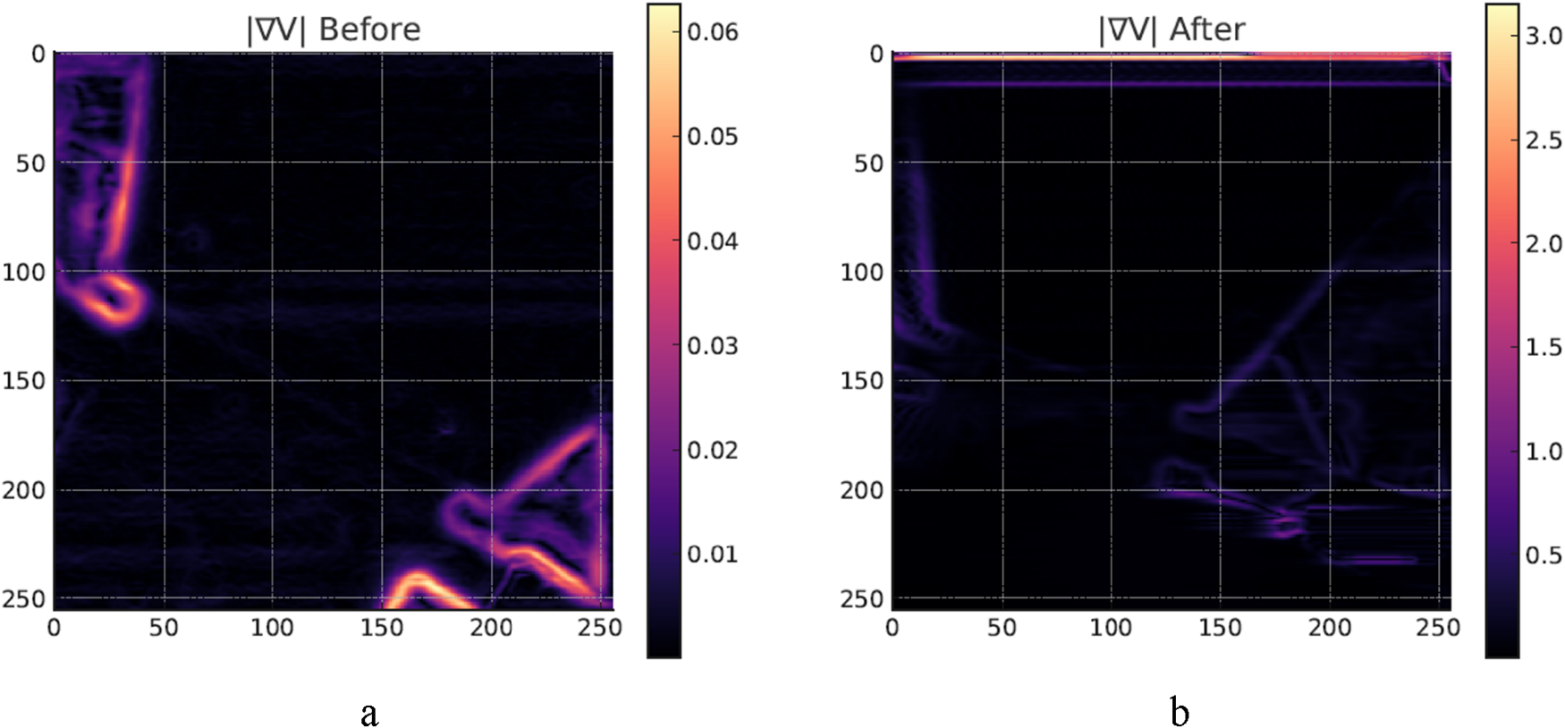
KPFM displaying spatial gradient maps (a) before and (b) after water exposure.

The presented CPD maps are representative of repeated measurements performed on multiple regions of the same device and on several devices, which showed consistent spatial trends before and after water exposure. All measurements were performed after thermal and mechanical stabilization of the system. No significant CPD offset drift was observed within the acquisition time of individual scans.

This measurement reveals localized regions with enhanced surface potential gradients near the edges of the device structures, indicating spatially inhomogeneous charge redistribution. These features may be influenced by heat treatment and contact proximity effects. After water exposure, the potential contrast becomes more uniformly distributed across the graphene surface, suggesting a more homogeneous adsorption-induced charge screening, which can contribute to the observed decrease in resistivity.

KPFM alone can show spatial work function shifts with humidity,^17,18^ but does not identify the chemical species responsible for those shifts. FTIR provides that chemical identification, enabling the attribution of observed CPD/work function changes to specific adsorbate exchanges and oxidation chemistry. Combining these techniques clarifies why humidity can sometimes decrease resistance in contaminated, ambient samples while increasing resistance on otherwise pristine surfaces. Before humidity exposure, the graphene channel exhibited an average work function of 5.31 ± 0.09 eV, while the device edges showed slightly higher values with an average of 5.45 ± 0.09 eV. After humidity exposure, the work function decreased in both regions. The graphene channel decreased to 3.72 ± 0.84 eV, corresponding to a reduction of 1.59 eV (∼30%). In contrast, the edge regions exhibited a significantly stronger change, with an average work function of 1.51 ± 4.06 eV, corresponding to a decrease of 3.94 eV (∼72%). These results indicate that the device edges are considerably more sensitive to humidity exposure than the graphene channel, which may be attributed to enhanced adsorption of water molecules and the presence of defects or exposed interfaces at the edges, leading to stronger modifications of the local electronic properties. It correlates with previous reported results which reported intentionally creation of defected graphene.^34^

To keep consistency with the electrostatic analysis, gate-dependent transfer characteristics (in Figure 8a,b) for a single-layer sample with channel dimensions of 50 × 30 μm (*W* × *L*) were measured before and after water vapor exposure as supporting electrical characterization. After humidity exposure, a systematic shift of the Dirac point toward higher gate voltages is observed. The shift is reproducible for all applied drain biases (600–1000 mV), indicating adsorption-induced electrostatic modulation of the graphene channel. Physically, this positive gate-voltage shift corresponds to effective p-type doping of graphene, which is attributed to adsorption-related charge transfer and dipolar gating effects associated with water molecules and oxygen-containing surface groups. This behavior is consistent with the KPFM-observed surface potential redistribution and work function modification, providing an independent electrostatic validation of the humidity-induced charge redistribution. The transfer curves also show a slight broadening of the minimum conductivity region after water exposure, suggesting modified scattering conditions and enhanced screening of charged impurities. These effects are consistent with the transport trends observed in the subsequent chemiresistive resistance measurements. Transfer curves shown in Figure 8 represent typical device behavior. Although averaging was not performed for this type of characteristic, the observed gate-voltage shift was reproducible across the measured device set.

**Figure 8:**
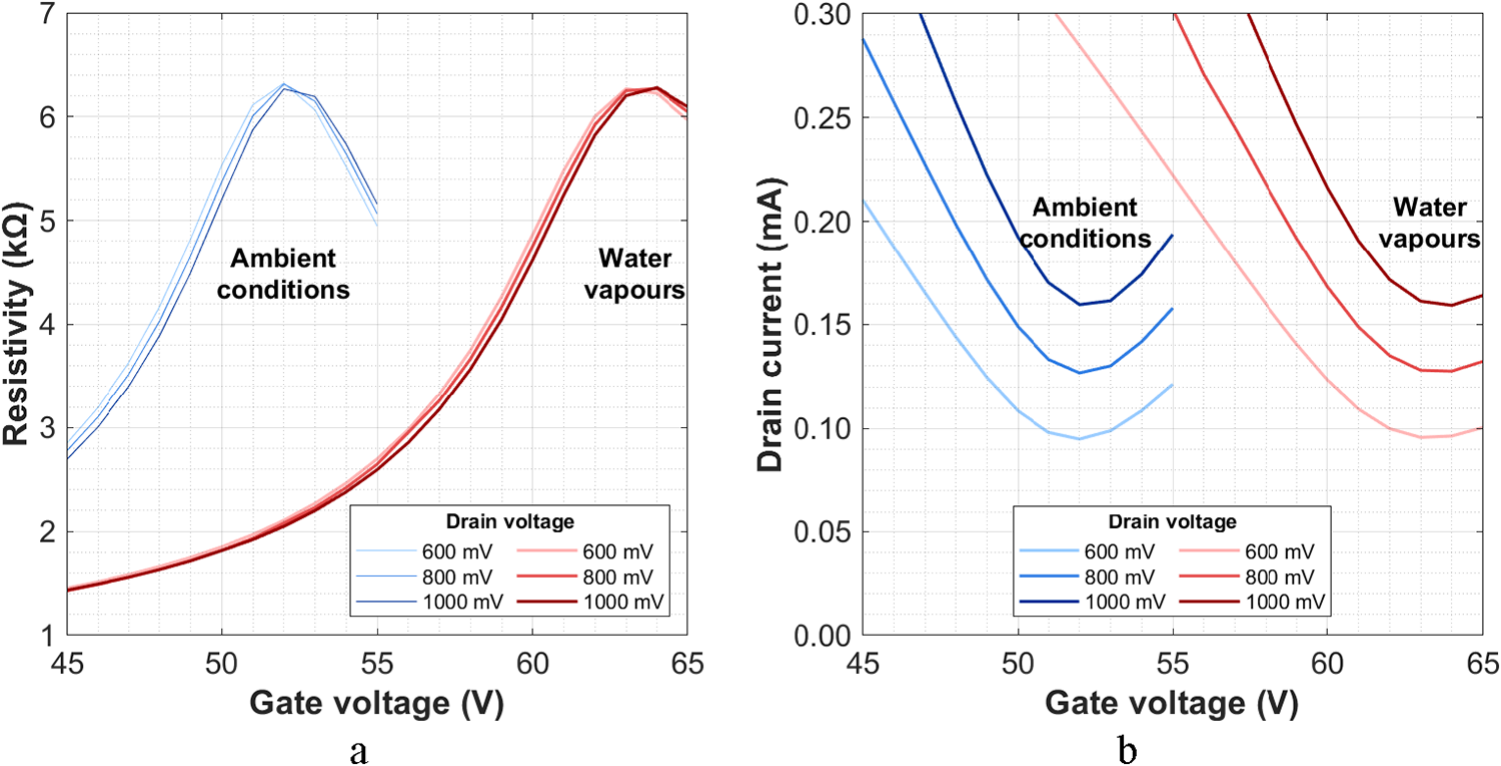
Gate-dependent transfer characteristics of the graphene device measured under ambient conditions and after water vapor exposure: (a) resistivity versus gate voltage, (b) drain current versus gate voltage, measured at different drain biases (600–1000 mV). A systematic shift of the Dirac point toward higher gate voltages is observed after humidity exposure.

The sample sensor has been measured as a resistor before and after exposure to water, and *I*–*V* characteristics have been taken. One sample (specifically one with the size of the channel of 40 μm) broke midmeasurement of the water treatment, therefore the after-water results for this specific sample are not included. This attrition rate continued with repeated measurements and bonding corrections, and as the samples were subjected to numerous measurements of different types, more sensor structures were deemed unfit for measurement. This affected the total set of measurements, but a minimum of three different measurements were taken each time, where possible. The *I*–*V* characteristics of all tested devices (Figure 9a) remain strictly linear within the applied voltage range (−1 V to +1 V), both before and after water vapor exposure. This confirms ohmic transport behavior and indicates that the measured resistance changes are not influenced by voltage-dependent transport artifacts or contact nonlinearity. Forward and backward voltage sweeps overlap within experimental uncertainty, indicating no measurable hysteresis in the investigated voltage range. This confirms that the observed resistance changes originate from adsorption-induced surface effects rather than from charge trapping or electrical instability during voltage cycling. With increasing channel width and while keeping the same channel length the overall resistance of the sample sensors fell following exponential function progress. The same can be estimated for the difference in resistance between clean and water-treated sample, with the exception of one sample with 100 μm channel size. To quantify the relative magnitude of the humidity-induced compensation effect across devices with different channel widths, the normalized resistance change (Δ*R*/*R*_0_ × 100) was calculated and is shown in Figure 9c. The results indicate that the relative resistance change remains comparable across channel geometries, suggesting that the compensating effect is primarily governed by surface adsorption processes rather than geometric scaling. The normalized resistance change ranged from approximately 5% to 50% across the investigated channel widths, with both expectations and trend suggesting higher change in resistance with increasing channel width. While the relative resistance change generally increases with channel width, some samples deviate from this trend. These deviations are attributed to variations in contact resistance, local defect density (as supported by Raman mapping) and nonuniform adsorption, which can dominate the electrical response in individual devices. Similar device-to-device variability has been reported previously for graphene FETs operating under environmental conditions, where local defects and contact defects can outweigh simple geometric scaling.^35–39^

**Figure 9:**
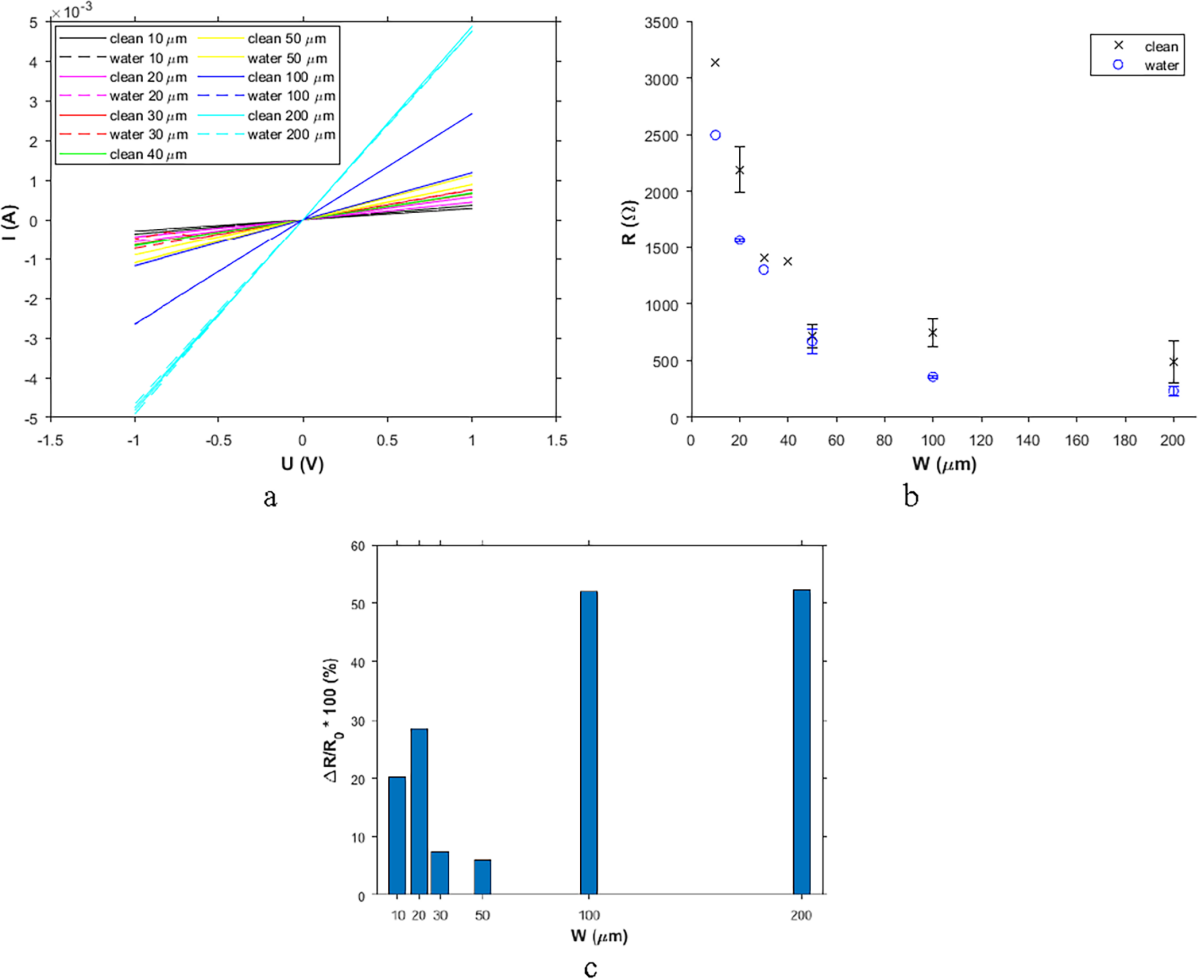
*I*–*V* characteristics of graphene-based devices before and after water vapor exposure, showing (a) linear and hysteresis-free behavior, (b) dependence of the resistance on channel width of different samples before and after exposure and (c) normalized resistance change (Δ*R*/*R*_0_ × 100) as a function of channel width, where *R*_0_ corresponds to the resistance measured under clean dry conditions.

Although the relative resistance change increases with channel width, this dependence is not expected to follow a strict exponential or logarithmic law, which Figure 9c might suggest. In an ideal graphene sensor device, channel resistance scales inversely with width, while contact resistance remains largely independent. The observed nonlinear trend therefore reflects a crossover from contact-limited to channel-limited transport, rather than an intrinsic shape of the dependence.^39–41^

A measurement in very clean environment has been performed to compare with the environmentally contaminated samples of the rest of the paper. The samples were cleaned under high temperature and then immediately moved to vacuum, where their surface was additionally purified by an electron beam (see the Materials and Methodssection for more details). This provided as clean of a graphene surface as was possible. Water vapor was introduced in small amounts, increasing the pressure of the vacuum chamber, and allowing the water to interact with the sample surface. The electric measurement from this process can be seen in Figure 10. The graph shows a slow increase in resistance with the introduction of humidity, with the occasional spikes that represent the opening of the valve, after which the signal stabilizes again. This slow resistance increase corresponds with the theoretical assumptions about pristine graphene conductivity,^42,43^ that any adsorption of contaminants or pollution of the surface would only hinder the charge carrier mobility and lead to decrease in conductivity. After removal of water vapor and subsequent vacuum annealing, the resistance returned to its initial baseline value within approximately 30 min, demonstrating that the humidity-induced response is reversible and does not lead to permanent device degradation. This relaxation behavior is consistent with thermally assisted desorption of physisorbed water molecules and weakly bound surface adsorbates from the graphene surface.

**Figure 10:**
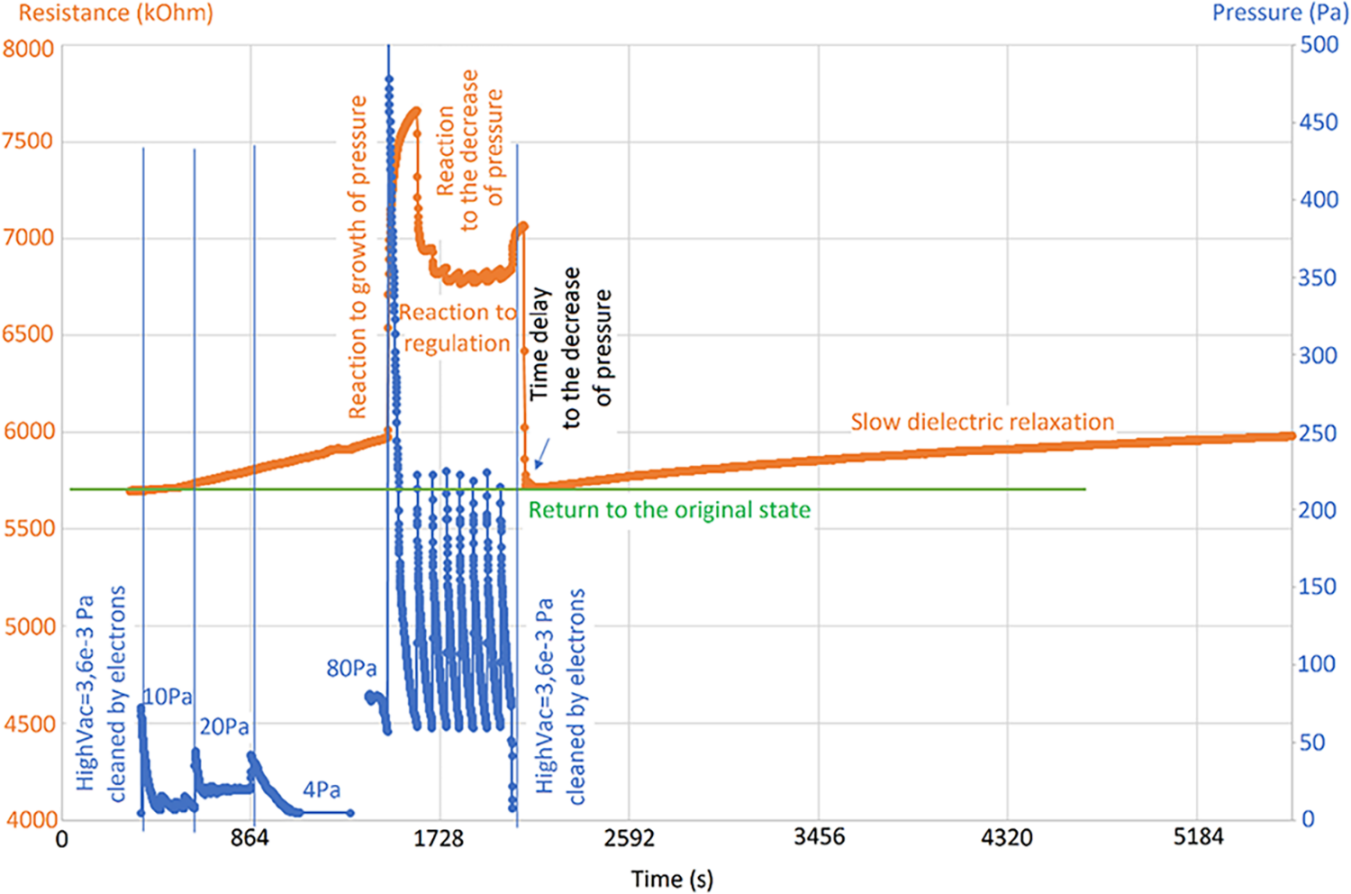
Dynamics of H_2_O detection under controlled pressure.

The observed dynamic resistance response and recovery behavior demonstrate that the device is capable of real-time monitoring of humidity-induced surface interactions. The reproducible response and reversible baseline recovery indicate suitability for repeated sensing cycles, which is essential for practical environmental and gas-sensing applications. From an application perspective, the demonstrated real-time resistance response, combined with stable ohmic behavior and reversible baseline recovery, indicates that graphene-based chemiresistive devices can be used for continuous environmental monitoring. The ability to track adsorption and desorption processes in real time enables detection of transient humidity fluctuations and surface contamination dynamics, which are relevant for air-quality sensing, wearable electronics, and low-power environmental sensor platforms.

To place the performance and scope of the present device in context, a comparison with representative graphene-based humidity sensing reports is summarized in Table 1.

**Table 1:** Comparison of Graphene-Based Humidity Sensors and Related 2D Material Sensing Platforms Reported in the Literature

Material	Sensing range	Key metric	Remarks
CVD graphene	Controlled RH (0–30%)	Simultaneous KPFM + transport correlation, surface charge propagation	KPFM humidity mapping^17^
CVD graphene	Wide RH (∼10–95%)	resistance change, fast response	example resistive graphene humidity sensor^44^
Graphene oxide (reduced GO sensor)	Controlled RH (1–96%)	Resistive change per % RH	Flexible humidity sensor based on chemically reduced graphene oxide^45^
2D FET sensors	broad overview	sensor trends	review of FET sensors and principles^46^
various graphene materials	broad overview	overview humidity values	review on graphene humidity sensors^47^

As shown in Table 1, most previous studies primarily report electrical response metrics, whereas the present work combines electrical measurements with multimodal surface and chemical characterization, enabling direct correlation between adsorbate exchange, work function modification and resistance response.

The data obtained from the environmental measurements go against both the purified vacuum data, and the theoretical expectations of the graphene sensor in ideal conditions. This is likely to the contamination of the sample. Under ideal circumstances, any impurity on the pristine graphene would result in an increase in resistance (as is supported by the presented measurement). Because the environmental measurements were taken without any attempts to stabilize or purify the environment, the surface of the sample is necessarily heavily contaminated with elements present in the air, regardless of any level of workplace cleanliness. These surface contaminants increase resistance. When water is bound to these contaminants, the defects undergo several processes, including their oxygenation and stabilization by H_2_O,^48,49^ which results in lowering of the resistance-increasing effect these contaminants originally had, so that the overall resistance starts to approach the pristine graphene state. As the contamination will always be present in an environmental measurement, however, it will never completely reach or overtake the ideal state and can only approach it.

To explain the stark divergence between the vacuum-annealed behavior (Figure 10) and the ambient measurements, our contamination-aware model suggests that water adsorption affects carrier transport through at least two competing mechanisms. In a pristine state, water acts purely as a scattering center, increasing resistance as theory predicts. However, in contaminated ambient conditions, it simultaneously replaces stronger pre-existing adsorbates (like CO_2_ and other airborne hydrocarbons), reducing local potential barriers and increasing carrier mobility, as evidenced by both the reduction in work function (Figure 6) and the homogenization of the electric field gradients (Figure 7). However, the global resistance response is governed by the balance between mobility enhancement and charge trapping at defects or functional sites created during oxidation.^50,51^

From a physical perspective, the observed mobility enhancement can be explained by dielectric screening and impurity compensation effects induced by water adsorption. Adsorbed H_2_O molecules form a polar surface layer that increases the effective local dielectric constant at the graphene interface, thereby reducing the Coulomb potential of charged impurities and lowering long-range scattering rates. At the same time, FTIR evidence of CO_2_ displacement indicates partial removal of strongly binding adsorbates that act as charged scattering centers. Together, these effects effectively reduce the impurity density experienced by charge carriers, particularly in p-type graphene regions where charged impurity scattering is dominant. As a result, the effective carrier mobility increases despite the presence of additional adsorbates, consistent with the KPFM-observed reduction in work function and homogenization of surface potential.^52–54^ Defects are localized mainly near the contacts, but resistance modulation originates primarily from the graphene channel. Contact regions contribute only secondary variability, indicating minimal influence on overall device behavior. The observed modulation is driven by adsorption-induced changes in the graphene channel rather than contact-limited effects.

Water-induced oxidation creates defect-associated trap states, which explain the mobility enhancements coexistence with nonmonotonic resistance behavior.^55^ These effects are consistent with reports of humidity-induced trap states in graphene-based transistors.^56,57^ Therefore, while water adsorption leads to a local improvement in carrier transport, the overall resistance trend reflects the superposition of mobility enhancement and charge trapping, particularly under environmental conditions where contaminants are present. No measurable hysteresis was observed because the device response was recorded under equilibrium conditions, with KPFM performed as stabilized surface-potential maps. The complete recovery of baseline resistance and the absence of hysteresis in dynamic electrical measurements indicate that humidity-induced adsorption–desorption is reversible and not controlled by slow trap-mediated processes.

While the adsorption/desorption dynamics of H_2_O and CO_2_ were not directly quantified in terms of absolute surface coverage or rate constants, they were inferred through the relative change in FTIR peak intensities and the corresponding real-time resistance evolution. In particular, the reduction of the CO_2_ peak around 2360 cm^–1^ after water exposure (Figure 4) correlates with the decrease in resistance observed under ambient conditions and with the homogenization of surface potential in KPFM (Figures 5–7). Conversely, during the vacuum experiments (Figure 10), the gradual increase in resistance correlates with increasing humidity, consistent with adsorption-driven scattering on otherwise pristine graphene. Although dynamic quantification was not possible with the current instrumentation, the observed correlations demonstrate a link between gas displacement and electrical response.

Raman mapping revealed a minor structural disorder localized near the metal contacts, which can influence carrier injection and local scattering. These defects act as a localized charge trapping sites, but their spatial confinement means that their contribution to the overall humidity response is secondary compared to the widespread interaction of water with surface adsorbates on the graphene channel. KPFM mapping (Figures 5–7) shows that after water exposure, the redistribution of surface potential occurs across the entire graphene area, not only near the contacts, indicating humidity-induced changes dominating over contact-related imperfections. Because resistance appears to scale with channel width (Figure 9) rather than defect density, the humidity sensitivity is likely to be governed primarily by adsorption on the channel surface, with the edge defects modulating charge injection only locally.

The influence of outer conditions could be mitigated by encapsulation,^58^ heat-reconditioning^59^ of the sensor or contact/edge protection^36^ of the graphene channel device, whereas if the environmental interaction is desirable, a controlled functionalization^60–62^ or patterned hydrophilicity^63^ could be used instead.

## 4 Conclusions

The combined structural, spectroscopic, and electrical analyses presented in this study demonstrate the complex influence of water vapor on graphene-based sensor device. While vacuum experiments validated the pristine graphene models (confirming a resistance increase upon water adsorption), environmental measurements revealed the opposite trend. This divergence emphasizes the necessity of a contamination-aware model: under ambient conditions, water does not merely act as a scatterer but actively mitigates the resistance-enhancing effects of pre-existing surface contaminants through adsorbate exchange.

Under ambient conditions, water vapor exposure resulted in a consistent resistance reduction across the investigated devices, with a normalized decrease of approximately 5–50% depending on the channel width (Figure 9c). This quantitative trend confirms the robustness of the compensating effect and demonstrates that water adsorption partially restores graphene’s intrinsic conductivity by mitigating the resistance-increasing influence of surface contaminants. The magnitude of this effect is consistent with the KPFM-observed reduction in work function and homogenization of surface potential, which indicate enhanced carrier mobility due to screening of charged impurities and adsorbate displacement. This divergence is attributed to the presence of surface contaminants, which interact with water molecules to reduce their resistance-enhancing effect and partially restore graphene’s intrinsic conductivity. Spectroscopic results confirmed that water molecules displace adsorbed CO_2_ and induce localized oxidation processes, while KPFM mapping revealed charge redistribution and a reduction in the work function consistent with improved carrier mobility. Localized contact defects influence the injection barrier and may introduce minor trapping, but their effect on humidity sensitivity is limited, compared to adsorption-driven changes over the channel surface. These findings show that contamination and fabrication induced disorder must not be dismissed when considering graphene properties in general, and response to water vapor in particular. In contrast to prior KPFM/transport reports, the combination of FTIR, KPFM, Raman and electrical measurements provide a chemical identifier of adsorbate exchange (CO_2_ and H_2_O) together with spatial potential mapping and macroscopic electrical comparison between vacuum-cleaned and ambient samples, thus revealing the dual role of water as both a contaminant stabilizer and a source of trap-forming oxidation. This integrated correlation is the primary contribution of this study and closes an experimental gap in humidity graphene research. This study, however, also has several limitations that should be addressed in future work. First, adsorption and desorption dynamics were inferred from static FTIR spectra and electrical measurements, but real-time quantification of surface coverage was not performed. Second, while ambient contamination likely influenced conductivity, the chemical identity of these contaminants was not analyzed in detail. Additional physicochemical surface characterization techniques, such as X-ray photoelectron spectroscopy (XPS) or in situ spectroscopic methods, would further improve identification of surface functional groups and adsorbate bonding states and will be considered in future studies. Third, only two environmental conditions were examined (vacuum-cleaned an ambient), without systematic testing across controlled humidity levels, due to the absence of a dedicated humidity-controlled environmental chamber. Fourth, the device architecture was limited to monolayer, CVD-grown graphene, and generalization to other forms of graphene or substrates would require additional validation. Finally, long-term stability, cycling behavior and baseline drift were not investigated. Future research should therefore focus on in situ spectroscopic monitoring, controlled humidity series, broader device architectures and extended durability testing. The demonstrated real-time response, reversibility, and device stability further highlight the applicability of the proposed graphene-based sensing platform for continuous humidity and environmental monitoring applications.

## Data Availability

10.5281/zenodo.18929113.
